# The effective function of circular RNA in colorectal cancer

**DOI:** 10.1186/s12935-021-02196-0

**Published:** 2021-09-17

**Authors:** Mandana Ameli-Mojarad, Melika Ameli-Mojarad, Mahrooyeh Hadizadeh, Chris Young, Hosna Babini, Ehsan Nazemalhosseini-Mojarad, Maziar Ashrafian Bonab

**Affiliations:** 1 Department of Biology, Faculty of Basic Science, Kharrazi University, Tehran, Iran; 2 School of Medicine, University of Sunderland, City Campus, Chester Road, Sunderland, SR1 3SD UK; 3 Institute of Health & Life Sciences, De Montfort University, Leicester, UK; 4 Department of Cell & Molecular Biology, Faculty of Science, Tehran University of Medical Science, Tehran, Iran; 5 Gastroenterology and Liver Disease Research Center, Research Institute for Gastroenterology and Liver Diseases, Shahid Beheshti University of Medical Sciences, Tehran, Iran

**Keywords:** Circular RNA, Colorectal cancer, Long non-coding RNA, Noncoding RNA

## Abstract

Colorectal cancer (CRC) is the 3rd most common type of cancer worldwide. Late detection plays role in one-third of annual mortality due to CRC. Therefore, it is essential to find a precise and optimal diagnostic and prognostic biomarker for the identification and treatment of colorectal tumorigenesis. Covalently closed, circular RNAs (circRNAs) are a class of non-coding RNAs, which can have the same function as microRNA (miRNA) sponges, as regulators of splicing and transcription, and as interactors with RNA-binding proteins (RBPs). Therefore, circRNAs have been investigated as specific targets for diagnostic and prognostic detection of CRC. These non-coding RNAs are also linked to metastasis, proliferation, differentiation, migration, angiogenesis, apoptosis, and drug resistance, illustrating the importance of understanding their involvement in the molecular mechanisms of development and progression of CRC. In this review, we present a detailed summary of recent findings relating to the dysregulation of circRNAs and their potential role in CRC.

## Background

Colorectal cancer (CRC) is one of the most common malignancies ranking third in the incidence and second in mortality among other cancers in the world. The global incidence of CRC is increasing, with approximately 3640 deaths and 17,930 new cases in 2020 [1, 2]. The exact mechanisms underlying CRC development remain unknown, however, risk factors that are strongly related to CRC include genetics, diet, tobacco smoking, heavy alcohol consumption, inactive lifestyle and age, where > 50 is a significant risk factor for CRC. However, recent evidence has also detected an increased risk for young adults [3]. Clearly the disorder is multifactorial in nature, with no common identifiable predictor of pre-disposition [4]. Here, we will review the molecular evidence to date.

Genetic and epigenetic alterations have both been found in CRC patients; changes in chromosomal copy number, aberrant gene methylation, and dysregulated gene expression, including tumor suppressor genes such as APC, BRAF, DCC, TP53, SMAD4, SMAD2, oncogenes such as KRAS and NRAS, and DNA repair genes including MLH1 and MSH6 [5, 6].

Dividing these mutation types into functional pathways broadly identifies three separate mechanisms: Chromosomal instability, which is the most common cause of genomic instability in CRC, significantly linked to alterations in APC and KRAS genes [7, 8]. In hereditary and sporadic colorectal cancer, microsatellite instability (MSI) is another key pathway. Germline mutation in one of the DNA mismatch repair genes, MLH1, MSH2, MSH6, or PMS2 leads to hereditary nonpolyposis colorectal cancer (HNPCC), while MSI in sporadic colorectal cancer is predominantly due to hypermethylation of the MLH1 promoter and sometimes sporadic mutations [9]. Defects in the mismatch repair mechanisms can also lead to MSI status [10]. A third pathway is via epigenetic alteration. CpG island methylator phenotype (CIMP) differences can result in changes in gene expression or function without changing the DNA sequence of that particular gene [11]. Taken together; these three pathways indicate the genetic heterogeneity of CRC.

CRCs are classified into 4 subtypes: CMS1-CMS4 with different clinical and biological characterizations [12]. Despite recent advances in our knowledge of signaling pathways involved in CRC, chemo- and radiotherapy resistance remains the most significant hurdle in CRC treatment. Therefore, a novel methodology for improved early diagnosis is essential. Non-coding RNAs (ncRNAs) play important roles in the regulation of chemo-and radio resistance of CRC [13]. Thus, ncRNAs could serve as targets for the development of new therapeutic strategies for drug and radiation resistance in CRC [14, 15]. circRNAs are a significant facet in ncRNAs biology, thus understanding of the role of circRNAs in CRC progression is pivotal to identifying new diagnostic, prognostic and predictive biomarkers for CRC [16]. In this review, we summarize the potential clinical implications of human circRNAs in CRC, for use as predictive biomarkers and/or therapeutic targets.

## The non-coding RNAs

The majority of the human genome (~ 90%) is transcribed as ncRNAs, which contain multiple classes of RNAs with various lengths [17]. Many studies have identified functional roles for ncRNAs, in various physiological and pathological processes, such as diabetes, cardiovascular disease, and cancer [18–20]. Classes of short ncRNAs include microRNAs (miRNAs), small interfering RNAs (siRNAs) and short piwi-interacting RNAs (piRNAs), meanwhile, linear lncRNAs (long non-coding RNAs) and circular RNAs are both classed as long noncoding RNAs [21]. circRNAs, however, are a new class of long ncRNAs, processing largely from exotic or intronic sequences, and are remarkably unique in structure and chemical characteristics compared with linear RNAs. circRNA biogenesis is based on the back‐splicing process, and closed 5-3ʹ ends negate degradation by RNA exonuclease or RNase R [22]. Classification of circRNAs is largely based on sequence origin, where subgroups include the circular intronic RNAs (ciRNAs), the exonic circRNAs (EcircRNAs), and exon–intron circRNAs (EIciRNAs) [23]. EcircRNAs, which predominantly exist in the cytoplasm, comprise the majority of all circRNAs. EcircRNAs can be formed by three different mechanisms, including lariat-driven circularization, RNA-binding protein (RBP)-driven circularization, and back splicing. EIciRNAs however, are formed only by back splicing of ciRNAs, which depends on a 7-nt GU-rich element and an 11-nt C-rich element, important in escaping debranching and exonucleolytic degradation [23, 24]. circRNAs have relatively stable structure and show tissue-specific expression, also displaying developmental stage regulation, with evolutionary conservation among species [25].

## Functions of circRNAs

**Fig. 1:**
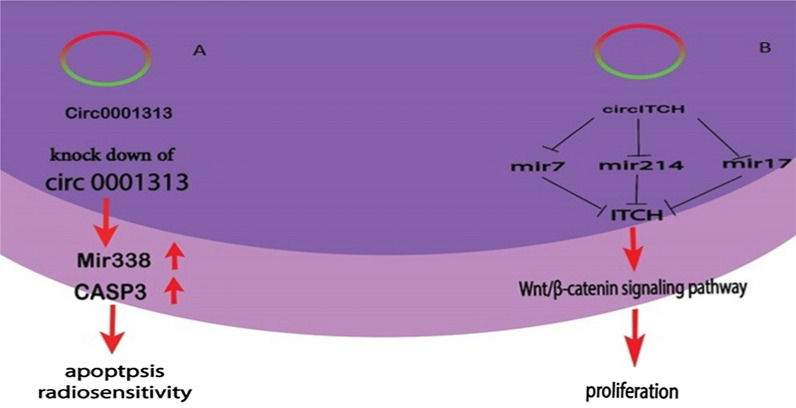
(A) Circ0001313 was found to be the most significantly upregulated circular RNA in CRC. where it can sponge miR-338 and affect apoptosis radiosensitivity in CRC. (B) Circ-ITCH is overexpressed in colorectal cancer and it can develop proliferation by sponging miR7, miR214, miR17 via Wnt/ β-catenin pathway signaling

circRNAs have regulatory roles in gene expression by sponging miRNAs, competing with other RNAs for binding to miRNAs and RNA binding proteins (RBPs) to modulate the local concentration of RBPs and RNAs as part of the competing endogenous RNA (ceRNA) network [26]. circRNAcircRNACDR1as (ciRS-7), for example, which has more than 70 conserved binding sites for miR-7, and is highly expressed in human and mouse brains [27, 28]. SRY, which encodes both linear and circular RNAs, is involved in sex determination in testis development. circRNA SRY can control metastasis and invasion of tumor cells via sponging miR-138 [29, 30]. Another circRNA, known as CircITCH, plays similar roles as a miRNA sponge, via miR-7, miR-17, and miR214, to inhibit proliferation through the Wnt/β-catenin signaling pathway [31], which is illustrated in Fig. 1A.

Although circRNAs are considered to be non-coding RNAs due to lack of 5’-cap structure and 3’-polyadenylation tail, circRNAs have been shown to generate protein products in a cap-independent manner [32]. Interestingly, many circRNAs are sometimes translated, indeed using high-content genomic screening, Legnini et al. found Circ-ZNF609 can translate into a protein in a splicing-dependent and cap-independent manner [33]. Yang Y et al. discovered CircFBXW7, produced from the FBXW7 gene, encoding a novel 21-kDa protein FBXW7-185aa, which reduced the half-life of c-Myc by antagonizing USP28-induced c-Myc stabilization [34].

**Fig. 2:**
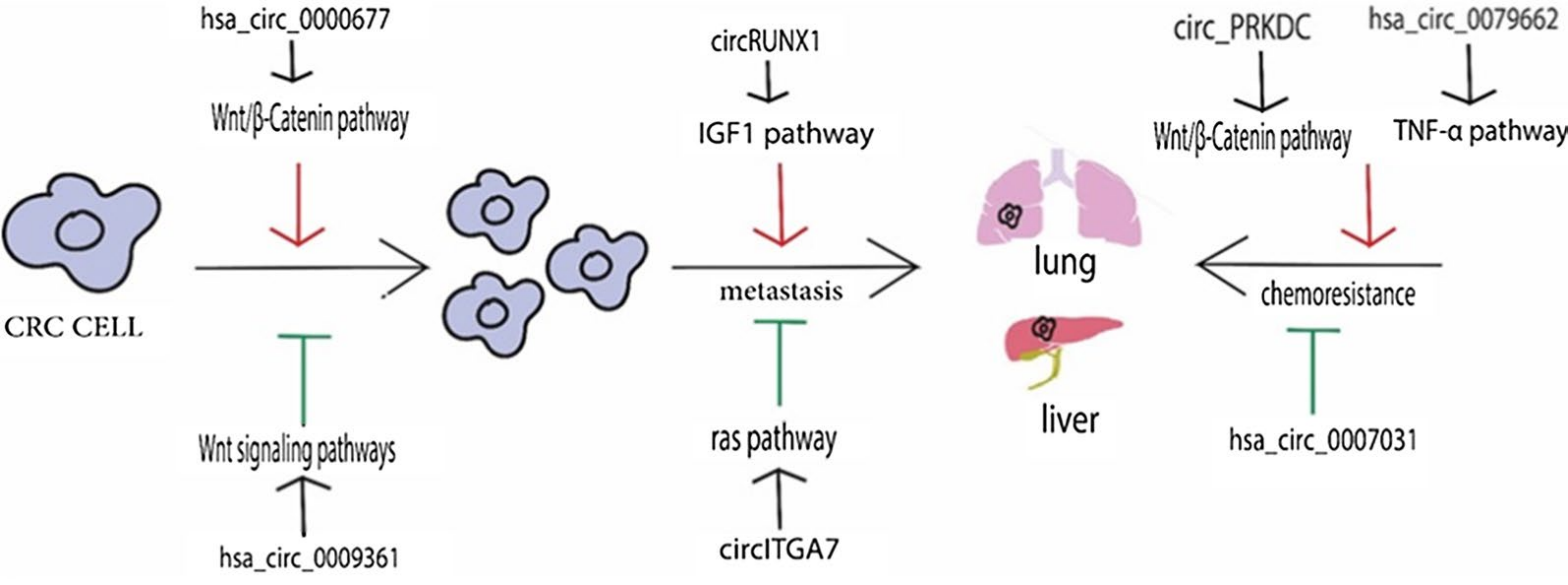
circRNAs and their targeted pathways in CRC including carcinogenesis, metastasis, and chemoresistance

The overall activities of circRNAs are intricately intertwined with RNA binding proteins, modulating the stability of mRNAs, regulating gene transcription, and translating proteins [35] and are involved in the regulation of cell proliferation, pluripotency and early lineage differentiation, epithelial-mesenchymal transition (EMT), cancer progression and chemoradiotherapy resistance, as shown in Fig. 2.

## Upregulation of circRNAs in CRC

Among all the validated aberrantly expressed circRNAs in colorectal cancer, upregulation of circRNAs more often associates with oncogenesis. Xia et al. found abnormally expressed circRNAs through CircRNA high-throughput sequencing, identifying Circ-0053277 as having the ability to sponge miR‐2467‐3p, and as being significantly upregulated in CRC tissues, where it facilitated CRC cell migration, proliferation, and epithelial‐mesenchymal transition [36]. Similarly, Li et al. identified CircVAPA as being upregulated in tissues and plasma, serving as a sponge for miR-101. Furthermore, they showed that the expression level of miR-125a was decreased in CRC cells, and CircVAPA knockdown repressed CRC cells cycle progression, invasion, and migration [37]. Knockdown of CircVAPA can also suppress CRC cell cycle progression, invasion, and migration by sponging miR-125a [38].

Yahang et al. found that Hsa_Circ_0026416 which was upregulated in CRC tissues and plasma, and has a key role in promoting the progression of CRC both in vitro and in vivo, may function as a ceRNA to sponge miR-346 [39].

Knockdown of another upregulated circRNA, CircACAP2 (hsa_circ_0007331), which was reported to be significantly upregulated in CRC tissues and colon cancer cells lines, suppressed proliferation and invasion by downregulating T lymphoma invasion and metastasis protein 1 (Tiam1) expression, through upregulated miR-21-5p expression (40). Another highly overexpressed circRNA in CRC is Hsa_circ_0136666, derived from the PRKDC gene, which can regulate proliferation and migration of CRC cells by sponging miR-136 [41].

## Downregulated circRNAs in CRC

As well as being overexpressed, other circRNAs are downregulated in CRC. Wang X et al. showed hsa_Circ_001988 was significantly downregulated in 31 matched colorectal cancer tissue samples, proposing this circRNA as a novel diagnosis potential biomarker in the CRC [42]. Geng Y reported hsa_Circ_0009361 to be significantly downregulated in both CRC tissues and derived cells. circRNA promoting the proliferation, epithelial-mesenchymal transition, migration, and invasion of CRC cells by sponging of miR-582. Conversely, overexpression of hsa_Circ_0009361 caused upregulation in the expression of adenomatous polyposis coli 2 (APC2) and blocked the activity of the Wnt/β-catenin pathway [43]. Circ-ITGA7, which sponges’ miR-370-3p to increase ITGA7 transcription–, through inhibition of RREB1 via oncogenic Ras has been shown to be down-regulated in CRC tissue samples [44]. Indeed, Circ-ITGA7 has also been shown to directly act as a tumor suppressor in CRC, with clinical features including cancer differentiation, lymph node metastasis, distant metastasis, and alterations in the TNM stage [45]. circRNA Circ-FBXW7 silencing was previously reported to enhance the proliferation, cell migration, and invasion of CRC cells in culture. In contrast, overexpression of Circ-FBXW7 significantly suppressed CRC cell proliferation, migration, and invasion. Similarly, Circ-FBXW7 silencing was also shown to stimulate tumor growth in SW480 and SW620 tumor models, whereas Circ-FBXW7 overexpression repressed tumor progression in the same system. This suggests that Circ-FBXW7 could serve as a target biomarker of CRC. Potential mechanisms have been proposed, including upregulated mRNA and protein expressions of NEK2 and mTOR, and diminished the PTEN expression (46). circRNACirc_021977 is another circRNA found to be down-regulated in CRC. Circ_021977 was shown to sponge miR-10b-5p, with a regulatory axis inhibiting proliferation, migration, and invasion in CRC via p21 and p53 [47]. Dysregulated circRNA expression in CRC is summarized in Table 1.

**Table 1:** The characteristics of dysregulated circRNAs in CRC

CircRNA	GENE Related miRNA	Expression	Targeted molecules/pathways	Function	References (DOI)		
circ_0007142	miR-455-5p	Up	SGK1	Regulates cell proliferation, apoptosis, migration, and invasion	[48]	2021	
hsa_circ_102049	miR-761, miR-192-3p	Up	FRAS1	Promoting liver metastasis	[49]	2021	
LONP2	Mir-17	Up	DGCR8	Prognostic predictor for anti-metastasis target	[50]	2020	
CircPTK2 (hsa_circ_0005273)		Up	binding to vimentin protein	Metastasis and may serve as a potential therapeutic target for CRC metastasis, Promote EMT	[51]	2020	
circPACRGL	miR142-39 506-3p	Up	TGF-β1	Promoted CRC cell proliferation, migration, and invasion, as well as differentiation	[52]	2020	
hsa_circ_0053277	miR-2467-3p	Up	MMP14	Facilitated the development of CRC accelerated cell proliferation	[37]	2020	
Hsa_circ_001680	miR-340	Up	BMI1	Enhance the proliferation and migration capacity of CRC cells	[53]	2020	
circSAMRCC1	miR-140-3p	Up	MMP-2, MMP-9, VEGF	Cell viability, migration, and invasion	[54]	2020	
CircHIPK3	miR-1207-5p	Up	FMNL2	Promote Cell Progression, migration, and invasion in CRC	[55]	2020	
circ-HIPK3	Mir-7	Up	FAK/IGF1R/EGFR/YY1	Promotes CRC growth and metastasis Prognostic	[56]	2020	
circHUWE1	miR-486	Up		Promotes Cell Proliferation, Migration, and Invasion	[57]		2020
circVAPA	miR-101	Up	CREB5	Promotes CRC cell proliferation, migration, invasion, and inhibit apoptosis	[38]	2020	
CircAPLP2	miR-101-3p	Up	Notch Signaling Notch1	Promotes proliferation and metastasis	[58]	2020	
circ-FARSA	miR-330-5p	Up	LASP1	Proliferation, migration, and invasion of CRC cells in vitro	[59]	2020	
CircAGFG1	miR-4262 and miR-185-5p	Up	WNT/β-catenin CTNNB1	Promote metastases	[60]	2020	
circ5615	miR-149-5p	Up	WNT/β-catenin pathway	Exerted oncogenic function	[61]	2020	
circular RNA 001,971	miR-29c-3p	Up	VEGFA	CRC cell proliferation, Invasion and angiogenesis	[62]	2020	
CircPRMT5	miR-377	Up	E2f3	Cell proliferation and migration	[63]	2020	
CircularRNA NOX4	microRNA-485-5p	Up	CKS1B	Promotes the development of colorectal cancer	[64]	2020	
circRAE1	miR-338-3p	Up	TYRO3	Promotes colorectal cancer cell migration and invasion	[65]	2020	
Hsa_circ_0079662		Up	TNF-α HOXA9	Induces the resistance mechanism of the chemotherapy drug oxaliplatin through the TNF‐α pathway	[66]	2020	
Hsa_circ_0026416	miR-346	Up	NFIB	Promotes proliferation and migration	[39]	2020	
circ_0136666	miR-383	Up	CREB1 proteins (HK2 and LDHA)	Accumulation on the proliferation and glycolysis and the promoting impact on the apoptosis of CRC	[67]	2020	
hsa_circRNA_102209	miR-761	Up		Promotes the growth and metastasis	[68]	2020	
Hsa_circ_0005963	miR-122	Up	PKM2	Chemoresistance. In vitro and in vivo studies	[69]	2020	
Circ TUBB				Interacting with smoking can enhance colorectal cancer risk	[70]	2020	
CircRNA_101951		Up	KIF3A	Promote migration and invasion	[71]	2020	
Circ-PNN hsa_circ_0101802)	miR-6833 miR-1301-3P	Up			[72]	2020	
circ-ABCC1 hsa_circ_0000677		Up	Wnt/β-catenin pathway	circ-ABCC1 was confirmed to facilitate CRC progression	[73]	2020	
CircFNDC3B	miR-937-5p	Up		circFNDC3B-enriched exosomes can inhibit angiogenesis and CRC progression	[74]	2020	
circ_0060745	miR-473,6	Up	CSE1L	Promotes Colorectal Cancer Cell Proliferation and Metastasis	[75]	2020	
circRUNX1	miR-145-5p	Up	IGF1 signaling	Promote Cell Growth Metastasis/Proliferation/ migration	[76]	2020	
circHOMER1	miR-138-5p	Up	HEY1	A decrease in glucose consumption Treated with lidocaine, indicating the inhibition of CRC cell viability mediated by lidocaine through suppressing aerobic glycolysis	[77]	2020	
Hsa_circ_0001806	miR-193a-5p	Up	COL1A1	Correlated with TNM stage, depth of invasion, lymphatic metastasis, and distant metastasis	[78]	2020	
circMAT2B	miR-610	Up	E2F1	Induces Colorectal Cancer Proliferation	[79]	2020	
circ_0000512	miR-296-5p/	Up	RUNX1	Cell Proliferation cell viability and colony formation	[80]	2020	
Circ_0056618	miR-206	Up	CXCR4 VEGF-A	Promoted cell proliferation, migration, and angiogenesis	[81]	2020	
CircRNA_0001946	MicroRNA-135a-5p	Up	EMT	A tumor promoter by activating the miR-135a	[82]	2020	
Hsa_circ_0038646	miR-331-3p	Up	GRIK3	Promotes cell proliferation and migration	[83]	2020	
Circ_0007031	miR-760	Up	DCP1A	Regulate the Growth and Chemoradiotherapy Resistance might play a positive role	[84]	2020	
Circ-PRKDC	miR-375/	Up	FOXM1 Axis and Wnt/β-Catenin	Circ-PRKDC enhanced 5-FU resistance in CRC	[85]	2020	
CircRNA UBAP2	Mir-199a	Up	VEGFA	Facilitated CRC progression	[86]	2020	
Hsa_circ_0000231	miR-502-5p	Up	MYO6	CRC progression It has a role in glycolysis	[87]	2020	
circGLIS2	miR-671	Up	NF-κB	Promotes colorectal cancer cell motility	[88]	2020	
Circular RNA CCDC66		Up	PI3KK	Apoptosis	[89]	2020	
circCCDC66	miR-3140	Up	autophagy	Promotes the tumorigenesis	[90]	2020	
circ-CCDC66	miR-33b/miR-93/	Up	DNMT3B/EZH2/ MYC/YAP1	Promotes CRC growth and metastasis	[91]	2020	
Hsa_circ_0128846	hsa-miR-1184	Up	YAP signaling	Promotes tumorigenesis	[92]	2020	
Hsa_circ_0007534	miR613 SLC25A22	Up	SLC25A22	Promote proliferation was correlated with tumor stage and lymph node metastasis	[93]	2020	
CircFAT1	miR-520b miR-302c-3p	Up	UHRF1	CRC cell proliferation, apoptosis, and glycolysis	[94]	2020	
CircFADS2		Up		Biomarkers of CRC	[95]	2020	
Circ-000166	miR-326	Up	LASP1	Cell growth and apoptosis in CRC cell lines	[96]	2020	
circ-ACAP2	Mir21-5p	Up	Tiam1	Promotes CRC cell proliferation, migration, and invasion	[49]	2020	
circ-ZNF609	miR-150	Up	Gli1	Promotes CRC cell migration	[33]	2020	
circ-NSD2	miR-199b	Up	5p/DDR1/JAG1	Promotes CRC metastasis	[97]	2020	
Circ-DENND4C	miR-760	Up	SLC2A1	Promote Migration and glycolysis	[98]	2020	
circ-Lgr4		Up	circLgr4-peptide/Lgr4/Wnt/β-catenin	Promotes CRC stem cell self-renewal, tumorigenesis and invasion	[99]	2020	
hsa_circ_000984	miR-106b	Up	CDK6	Promotes CRC growth and metastasis	[100]	2020	
Has _circ -140,388 (circHUWE1)	Mir486 .5p	Up	PLAGL2 IGF2 WNT- β CATENIN	Proliferation, migration, invasion,	[57]	2020	
Has-circ-0004680 circCCT3	Mir- 613	Up	CCT3 /WNT3/VGFR	Metastasis	[101]	2020	
Has _circ -001,900 circCAMSAP1	Mir328-5p Mir7	Up	E2F1 EGFR IGF1R CAMSAP1	Promotes CRC progression	[102]	2020	
hsa_circ_0007534		Up		Promotes proliferation and inhibits apoptosis	[93]	2021	
Has-circ- 0,007,843	Mir- 518-5p	Up	ARHGAP32	Migration, invasion,	[103]	2020	
circRNA_100876	miR-516b	Up		Inhibit proliferation and metastasis	[104]	2020	
CircRNA_0000392	miR-193a-5p	Up	PIK3R3/AKT	Promoter proliferation of CRC	[105]	2020	
circRNA_002144	miR-615-5p	Up	LARP1	Promotes growth and metastasis	[106]	2020	
Circ-Erbin	miR-125a-5p and miR-138-5p,	Up	4EBP-1	Promotes growth and metastasis of CRC	[107]	2020	
CircRNA 100,146	miR-149	Up	HMGA2	Promotes Colorectal Cancer Progression	[108]	2020	
circ-NSUN2		Up	IGF2BP2/HMGA2	Promotes CRC liver metastasis	[109]	2019	
circCCT3	Mir613	Up	VEGFA; WNT signaling	Contributes to metastases	[101]	2019	
Circ_0000218	miR-139-3p	Up	RAB1A	Promoted CRC proliferation and metastasis via	[110]	2019	
circFMN2	miR-1182	Up	hTERT	Cell proliferation and migration	[111]	2019	
Circ 32,883	Mir501-5p	Up	EmL5	Promote resistance to folfox	[112]	2019	
Circ ACC1		Up	c-Jun/AMPK	Promotes CRC cell fatty acid β-oxidation, glycolysis and growth	[113]	2019	
hsa_circ_102958	miR-585	Up	CDC25B	Promotes CRC tumorigenesis	[114]	2019	
Has- circ-101555	Mir 597-5p	Up	CDK6 RPA3	Promote progression	[115]	2019	
Has-circ-0079993	Mir 139-3p	Up	CREB1	Promotes CRC cell proliferation	[116]	2019	
Has-circ- PIP5K1A	Mir1273 Irf4 cdx2 ZIC1	Up		Promote progression CRC	[117]	2019	
hsa_circ_0055625	ITGB8	Up	miR-106b	Increases colon cancer cell growth was associated with pathological TNM stage and metastasis	[118]	2019	
hsa_circ_0136666	PRKDC SH2B1	Up	Mir136	Promote proliferation and invasion	[41]	2019	
hsa_circ_0073195	miR-199-b	Up	Ddr1 and Jag1 signaling	Promotes metastasis	[97]	2019	
hsa_circ_0071589	MIR-600	Up	Fat1 EZH2	Promotes carcinogenesis tumor growth, invasion, and migration	[119]	2018	
circRNA_100290	FZD4 SLC30A7 WNT/β-catenin	Up	Mir516b	Promotes colorectal cancer	[120]	2018	
Cirs7	miR-7	Up	EGFR and IGF1R	Promotes progression	[27, 121]	2017	
Circ0000504	Mir485-5p	Up	Tubgcp3 Stat3	Promote resistance to 5fu	[122]	2017	
hsa_circ_000984	CDK6	Up	Mir 106b	Promotes cells proliferation and metastasis	[100]	2017	
hsa_circ_0020397 (circBANP)	DOCK1 PD_L1 TERT	Up	Mir138	Can regulate CRC cell viability, apoptosis, and invasion	[123]	2017	
Circ-0001313	miR-3383p 33b5p 935p	Up	Ccdc66	Promote resistance to radiotherapy and 5fu	[124]	2019 2017	
Has-circ-001569	miR145	Up	ABC1 E2f5 BAG4	The regulator in cell proliferation and invasion	[125]	2016	
circ_0007142	miR-122-5p	Down	CDC25A	Proliferation, colony formation, migration, and invasion	[48]	2020	
CircCSNK1G1	miR-455-3p	Down	MYO6	Proliferation, migration and invasion cell growth and metastasis,	[126]	2020	
CircTADA2A	miR-374a-3p. MiR-374a-3p	Down	KLF14	Tumor suppressor in CRC	[127]	2020	
circ-SMAD7		Down		circ-SMAD7 could inhibit cell migration and invasion of CRC by suppressing the EMT process,	[128]	2020	
Circ_cse1l		Down	eIF4A3 PCNA	circ_cse1l inhibited the proliferation of CRC	[129]	2020	
ITGA5 circRNA	miR-107,	Down	FOXJ3	Act as a tumor suppressor in CRC	[130]	2020	
CircDDX17	miR-31-5p/	Down	KANK1	Tumor suppressor blocked CRC progression Strengthened chemosensitivity of CRC to 5-Fu	[131]	2020	
Hsa_circ_0137008	microRNA-338-5p	Down		Inhibited the progression of CRC	[132]	2020	
CircNOL10	miR-135a-5p; miR-135b-5p	Down	KLF9	Mediating proliferation, cell cycle, migration, and invasion	[133]	2020	
circ_0021977	miR-10b-5p	Down	P21; P53	Suppresses proliferation, migration, and invasion by CRC cells	[47]	2020	
circRNACBL11	YWHAE	Up	Mir6778-5p	Suppress cell proliferation	[134]	2019	
Circ. CDYL	c-Myc cyclin D1	Down	miR-150-5p/	Inhibits CRC cell growth and migration	[135]	2019	
circITGA7	ITGA7 REB1 Ras’s pathway ASXL1	Down	miR-370-3p mir-3187-3p	Inhibits colorectal cancer growth and metastasis	[44] [45]	2019 2018	
hsa_circ_0009361	Mir582-3p	Down	APC2/Wnt/β-catenin	Inhibits CRC progression	[43]	2019	
hsa_circ_0000523	METTL3 dKK1 WNT/β-catenin	Down	Mir-31	Correlated to the tumorigenesis- Proliferation	[136]	2018	
circITCH	DDX17 WNT/β-catenin	Down	miR-7, miR-17, miR-214	Proliferation ( −)	[31]	2015	

## circRNAs in predicting response to chemoradiotherapy

Targeted therapy, chemotherapy, and multiagent regimens, for example, FOLFIRI (5-FU and irinotecan) and FOLFOX (5-FU oxaliplatin) can be applied as the standard treatment of CRC. However, chemotherapy has its limitations, including toxicity, low response rates, unpredictable innate and acquired resistance mechanisms, and low tumor-specific selectivity [137]. Recent studies have shown that different ncRNAs such as circRNAs, may play important roles in the regulation of chemoresistance and affect the sensitivity of tumors to chemotherapy and radiotherapy through modification of various signaling pathways, including cell cycle, proliferation, apoptosis, and DNA damage repair [84, 112]. hsa_circRNA_0001313 is one of the upregulated circRNAs in radio-resistant CRC tissues. Inhibition of hsa_circRNA_0001313 induces radio-sensitivity, reduced cell viability, and increases caspase-3 activity and colony formation by negatively modifying miR-338-3p in CRC cells, which has shown in Fig. 1B [124]. Another recent study reported that CircDDX17 was down-regulated in CRC, and its overexpression induced inhibition of 5-Fu resistance, blocked tumor growth, and CRC progression via sponging miR-31-5p [131]. Interestingly, Circ-32883 was upregulated in CRC tissues and its overexpression was positively associated with chemoresistance through its potential action as a sponge for miR-501-5p. This miRNA binds to EML5 mRNA, inhibiting its expression. Thus, promoting resistance to FOLFOX therapy [112]. Other circRNAs related to chemotherapy resistance are summarized in Table 2.

**Table 2:** The characteristics of circRNAs in CRC as a chemotherapy resistance

CircRNA	GENE related miRNA	Expression	Targeted molecules/pathways	Function	References (DOI)	Year
Hsa_circ_0079662		Up	TNF-α HOXA9	Induces the resistance mechanism of the chemotherapy drug oxaliplatin through the TNF‐α pathway	[66]	2020
Hsa_circ_0005963	miR-122	Up	PKM2	chemoresistance. In vitro and in vivo	[69]	2020
Circ_0007031	miR-760	Up	DCP1A	Regulate the Growth and Chemoradiotherapy Resistance	[84]	2020
CircDDX17	miR-31-5p	Down	KANK1	Tumor suppressor Strengthened chemosensitivity of CRC to 5-Fu	[131]	2020
Circ-PRKDC	miR-375	Up	FOXM1 Axis and WBT/β-Catenin	Enhanced 5-FU resistance in CRC	[85]	2020
Circ-0001313	mir-3383p mir33b5p mir935p	Up	Ccdc66	Promote resistance to radiotherapy and 5fu	[124]	2019
Circ 32,883	Mir501-5p	Up	EmL5	Promote resistance to folfox		2019
Circ0007006	Mir300 653-5p 628-5p	Up		Promote resistance to 5fu	[122]	2017
Circ0000504	Mir485-5p	Up	Tubgcp3 Stat3	Promote resistance to 5fu	[122]	2017

## circRNAs as biomarkers for colorectal cancer

Through improvements in high-throughput sequencing, circRNA microarray, and chip analysis we now know circRNAs are differentially expressed in CRC, and certain circRNAs are involved in various biological processes such as proliferation, migration, invasion, and apoptosis. Due to the unique structure of circRNAs, which confers resistance to RNase and longer half-lives, they can therefore be potential candidates for diagnostic biomarkers. However, the underlying biological function of circRNAs requires further investigation [138, 139].

Several circRNAs have been proposed as useful therapeutic targets for CRC. For instance, hsa_circ_022382 which is derived from the human FADS2 gene is overexpressed in 200 CRC tissues, where CircFADS2 overexpression was positively associated with clinicopathological features. CircFADS2 expression may therefore be a promising biomarker for prognostic investigation in CRC patients [95]. In another study, hsa_circ_0026344 was shown to be significantly down-regulated in 32 CRC patients compared to paired adjacent non-tumorous tissues. The expression of hsa_circ_0026344 was correlated with tumor size and lymph metastasis. Functionally, circRNA-0026344 overexpression significantly suppressed CRC cell proliferation and colony formation as well as promoted apoptosis by regulating miR-21 and miR-31 levels [45]. Other circRNAs with biomarker potential are summarized in Table 3.

**Table 3:** circRNAs with Biomarker potential in CRC

CircRNA	GENE related miRNA	Expression	Targeted molecules/pathways	Function	References (DOI)	
Hsa_circ_0002320		Down		Noninvasive diagnostic blood biomarker for CRC prognosis	[140]	2020
circMBOAT2	miR-519d-3p	Up tissues serum	TROAP)	A novel tumor marker and regulates proliferation/migration	[141]	2020
hsa_circ_0060927		Up		Potential diagnostic markers	[142]	2020
circ-CCDC66	miR-33b/miR-93/	Up	DNMT3B/EZH2/ MYC/YAP1	Promoting CRC growth and metastasis	[91]	2020
circ_0005075		Up	Wnt/β-catenin pathway	Potential target for the prognosis biomarker	[143]	2020
Hsa_circ_0004831		Up	WNT and p53 signaling pathway	Prognostic biomarker	[144]	2020
hsa_circ_104916		Down		Prognosis biomarker Inhibiting CRC cell proliferation, migration, invasion, and inducing apoptosis	[145]	2019
hsa_circ_0004585		Up		Potential diagnostic biomarker for CRC	[146]	2019
hsa-circ-0004771		Up		Nvel potential diagnostic biomarker	[147]	2019
circ-PPP1R12A Has-circ- 000,423		Up	Hippo/YAP Prognosis	Prognostic biomarker Promoting pathogenesis and metastasis	[148]	2019
circ-MTO1		Down	WNT/β-catenin	Prognostic biomarker, Inhibiting cell proliferation and invasion	[149]	2018
hsa_circ_0001649	SHARE	Down		Novel diagnostic biomarker Expression level is closely associated with pathological differentiation	[150]	2018
Has _circ_ 14,717		Down	P16	Prognostic biomarker Inhibiting CRC cell proliferation, colony formation, and growth	[151]	2018
hsa_circ_0026344	miR-21/miR-31	Down		Prognostic biomarker Inhibiting CRC cell growth and invasion and induces apoptosis	[45]	2018
Has-circ-0000711		Down		Diagnostic Prognostic biomarker	[152]	2018
Cirs-7	CDR1 EGFR/RAF1/MAPK pathway	Up	Mir-7	Prognostic biomarker	[27]	2018
hsa_circ_0000567	SETD3	Down			[153]	2018
hsa_circ_001988	FBXW7	Down		Potential diagnostic biomarker	[42]	2015
hsa_circ_0003906		Down		Diagnostic biomarker	[154]]	2015

## circRNAs as therapeutic targets in colorectal cancer

**Fig. 3:**
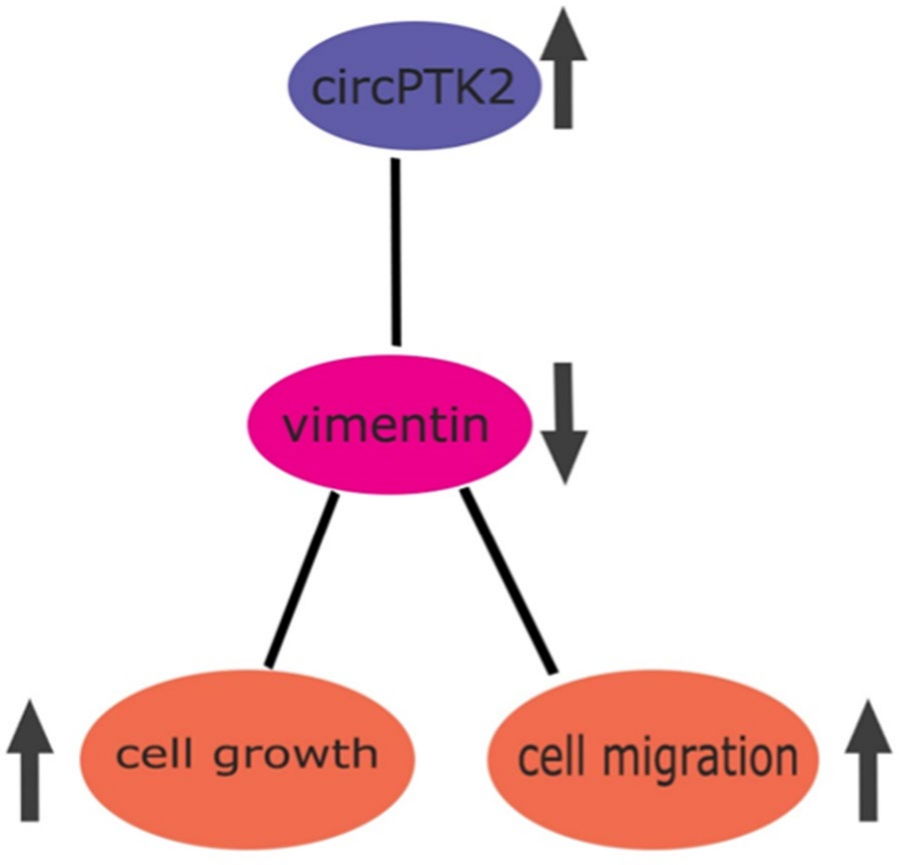
CircPTK2 is overexpressed in CRC tissues and is associated with tumor metastasis

Targeted therapy has been widely used in the clinic due to its excellent efficacy, and it can work on cancerous cells by directly inhibiting cell proliferation, differentiation, and migration [50]. Indeed, monoclonal antibodies, for instance, are currently an important player in targeted therapies [51]. circRNAs moderate drug resistance by sponging microRNAs both in traditional chemotherapeutic drugs, advanced targeted drugs, and immunotherapeutic drugs. For example, therapeutic targeting of ciRS-7 may become a promising strategy for colorectal cancer patients, since higher expression of ciRS-7 correlated with multiple clinicopathologic factors, such as advanced T-stage, lymph node, and distant metastasis, and ciRS-7 overexpression promotes the EGFR/RAF1/MAPK pathway by inhibiting miR-7 activity [121, 155]. Yang et al. indicated that high expression of circPTK2 positively correlated with poorer survival, showing CircPTK2 can bind to vimentin and promote EMT growth and metastasis in CRC cells, therefore ciRS-7 may become a therapeutic target for CRC metastasis [51]. The relation between circPTK2 in CRC is shown in Fig. 3.

Another highly expressed circRNA in CRC tissue is Circ_001680 which was observed to enhance the proliferation and migration capacity of CRC cells. Fluorescence reporter assays confirmed that circ_001680 alters the expression of BMI1 by targeting miR-340. More importantly, Circ_001680 was found to promote the propogation of cancer stem cells in CRC and induce resistance against Irinote by modifying the miR-340 target gene BMI1 n [53]. Safe and effective delivery of ncRNAs is a significant therapeutic paradigm for all cancers. Since unmodified oligonucleotides are not stable in circulation, modifications of oligonucleotides are essential to increasing efficacy and stability. Most current oligonucleotide therapies need an additional delivery system to achieve these desired biological effects. Several options need to be considered in selecting a delivery system, including stability, evasion of the innate immune system, avoidance of non-specific interactions with serum proteins, and non-target cells. One of the common strategies to increase the circulation time for therapeutic oligonucleotides is shielding the exterior of delivery vehicles with polyethylene glycol (PEG). This strategy may prevent the non-specific function of particles with immune cells and other non-target tissues. Although a variety of delivery systems has been developed in the laboratory, challenges remain in bringing the full potential of RNAi to clinical approaches [156]. circRNAs however, offer significant increases in stability over current strategies.

## Conclusions and perspectives

Following advancements in high-throughput sequencing, the field of circRNAs has attracted more attention and is currently an area of intense interest in the field of cancer research. circRNAs are an ideal biomarker in cancer, and are stably expressed in exosomes, blood, and saliva, where specific circRNAs have been indicated as promising prognostic or diagnostic biomarkers already.

Abnormal expression of circRNAs has been observed in a wide range of human malignancies and their dysregulation can alter gene expression networks, leading to dramatic changes in cell fates, including cancer initiation and progression. circRNAs can be both oncogenic and anti-oncogenic, so could potentially be utilized in the treatment and prognosis of colorectal cancer. Although recent advances on circRNAs have highlighted some interesting insights, much work remains to be done to translate circRNAs into clinical application for clinical patient benefit. Major hurdles include the development of an efficient siRNAs delivery system, and the assessment of safety and side effects, yet, clearly circRNAs have significant potential for the treatment and diagnosis of CRC.

## Data Availability

Data will be provided based on reasonable request.

## Abbreviations

ceRNA: Competing endogenous RNA
circRNAs: Circular RNAs
siRNA: Small interacting RNA
ncRNA: Noncoding RNA
HNPCC: Hereditary nonpolyposis colorectal cancer
ciRNAs: Intronic circRNAs
ecircRNA: Exonic circRNAs
ELciRNA: Exon–intron-circRNAs
miRNA: MicroRNA
RBP: RNA-binding protein
PEG: Polyethylene glycol
CRC: Colorectal cancer
